# H3K4 demethylase activities repress proliferative and postmitotic aging

**DOI:** 10.1111/acel.12166

**Published:** 2013-11-19

**Authors:** Stacy M Alvares, Gaea A Mayberry, Ebony Y Joyner, Bernard Lakowski, Shawn Ahmed

**Affiliations:** 1 Department of Genetics, University of North CarolinaChapel Hill, NC, 27599-3280, USA; 2 SPIRE Postdoctoral Fellowship Program, University of North CarolinaChapel Hill, NC, 27599-3280, USA; 3 Department of Biology, University of North CarolinaChapel Hill, NC, 27599-3280, USA; 4 Department of Natural Sciences, Fayetteville State UniversityFayetteville, NC, 28301-4298, USA; 5 Department of Neuroscience, Institut Pasteur75724 Paris Cedex 15, France

**Keywords:** *Caenorhabditis elegans*, cellular aging, chromatin, histone demethylase, life span

## Abstract

Homeostasis of postmitotic and proliferating cells is maintained by pathways that repress stress. We found that the *Caenorhabditis elegans* histone 3 lysine 4 (H3K4) demethylases RBR-2 and SPR-5 promoted postmitotic longevity of stress-resistant *daf-2* adults, altered pools of methylated H3K4, and promoted silencing of some *daf-2* target genes. In addition, RBR-2 and SPR-5 were required for germ cell immortality at a high temperature. Transgenerational proliferative aging was enhanced for *spr-5*; *rbr-2* double mutants, suggesting that these histone demethylases may function sequentially to promote germ cell immortality by targeting distinct H3K4 methyl marks. RBR-2 did not play a comparable role in the maintenance of quiescent germ cells in dauer larvae, implying that it represses stress that occurs as a consequence of germ cell proliferation, rather than stress that accumulates in nondividing cells. We propose that H3K4 demethylase activities promote the maintenance of chromatin states during stressful growth conditions, thereby repressing postmitotic aging of somatic cells as well as proliferative aging of germ cells.

## Introduction

Cellular aging has been attributed to the dysfunction of multiple maintenance mechanisms. As humans age, global changes in epigenetic mechanisms occur, which alters the regulation of gene expression (Bocklandt *et al*., 2011). Further, in the accelerated aging disorder Hutchinson–Gilford Progeria syndrome, nuclear lamin A defects lead to dramatic changes in epigenetic modifications prior to alterations in nuclear morphology associated with disease progression (Shumaker *et al*., 2006). Because epigenetic modifications are reversible and could represent attractive therapeutic targets, it is important to understand how such modifications influence aging (Stilling & Fischer, 2011).

An epigenetic mark that is strongly correlated with transcriptional activation is the methylation state on histone 3 lysine 4 (H3K4), which can be mono (Me1)-, di (Me2)-, or tri (Me3)-methylated. The methylation status of any nucleosome is determined by a balance of the activities of specific histone lysine methyltransferases (KMTs) and histone lysine demethylases (KDMs) (summarized in Allis *et al*., 2007). Two classes of enzymes remove lysine-methyl marks: amino oxidases (KDM1) and Jumanji C (JmjC) domain-containing proteins. KDM1 proteins only remove Me1 and Me2 in a reaction that requires the cofactor flavin adenine dinucleotide (FAD) (Hou & Yu, 2010). Demethylases containing the JmjC domain can catalyze the removal of all methylation marks (Huang *et al*., 2006; Tsukada *et al*., 2006), but different classes of JmjC proteins are only capable of removing a subset of methyl marks from specific lysine residues *in vivo* (Allis *et al*., 2007). The KDM5 class of JmjC proteins has specificity for H3K4. In humans, there are four KDM5 proteins, but many lower organisms possess only one member of this class (Allis *et al*., 2007).

A well-established system for studying the genetics and epigenetics of aging is the nematode *Caenorhabditis elegans*. Although H3K4 methylation can impact aging, KDM1 and KDM5 demethylases do so in opposing ways. The sole *C. elegans* KDM5 member, RBR-2, can remove both Me3 and Me2 marks from H3K4, but is more efficient at removing Me3 (Christensen *et al*., 2007). RBR-2 was originally identified as being transcriptionally down-regulated in long-lived *daf-2* insulin/IGF-1 signaling mutants, suggesting that H3K4 demethylation may promote aging (Lee *et al*., 2003). However, overexpression of *rbr-2* in the germline represses aging and extends lifespan, while the *rbr-2(tm1231)* deletion mutant is short-lived (Greer *et al*., 2010). Furthermore, reduction in function of components of the ASH-2 trithorax complex, which results in reduced levels of H3K4 trimethylation, increases lifespan, and this effect is suppressed by deficiency for *rbr-2* (Greer *et al*., 2010). Together, these results suggest that H3K4 methylation can be a pro-aging chromatin mark.

Opposing effects were observed when KDM1 demethylase activity was compromised. In *C. elegans*, there are three KDM1 genes. *amx-1* encodes the sole KDM1B enzyme, while there are two paralogous KDM1A enzymes encoded by the *spr-5* and *lsd-1* genes (Eimer *et al*., 2002; Jarriault & Greenwald, 2002). Animals with a reduced function for *lsd-1* have a longer life compared to wild-type strains (McColl *et al*., 2008; Maures *et al*., 2011). Thus, KDM1 and KDM5 enzymes may exert opposite effects on postmitotic aging by targeting enzyme class-specific substrates for H3K4 demethylation.

Although adult somatic tissue is postmitotic in *C. elegans*, aging of proliferating cells is relevant to organisms where somatic stem cell populations continue to divide throughout life. The germline is capable of maintaining itself in a nonaging pluripotent state over the generations, despite indefinite proliferation. Genes that promote proliferative immortality of germ cells have been identified in screens for *C. elegans mortal germline* (*mrt*) mutants that are initially fertile and become sterile after propagation for a number of generations, including several mutants that are defective for telomerase-mediated telomere replication (Ahmed & Hodgkin, 2000; Meier *et al*., 2006).

H3K4 methylation plays important roles in the germ cells of many organisms: H3K4 methylation is absent in the pole cells found in the Drosophila germline but accumulates with age, whereas removal of H3K4me2 occurs during the birth of germ cell precursors in *C. elegans* and remains absent until these cells begin to divide in the L1 larval stage (Schaner *et al*., 2003). KDM1 members play a role in this process, as mutations in *spr-5* could elicit fertility defects after many generations, and this was correlated with increasing retention of H3K4 methylation in germline precursor cells (Katz *et al*., 2009). *spr-5* elicited variable and progressive fertility defects, where high levels of sterility occurred after growth for 20 generations, an effect that was enhanced in *spr-5*; *amx-1* double mutants (Katz *et al*., 2009).

To characterize the role of H3K4 demethylases in the maintenance of somatic and germ cells, we examined the impact of *rbr-2* deficiency on somatic longevity in adults, as well as in germ cell maintenance as they proliferate over many generations, and in the maintenance of nondividing germ cells in long-lived dauer larvae. We also examined the function of *spr-5*. Both *rbr-2* and *spr-5* extended longevity in a manner that depended on reduced insulin/IGF-1 signaling, implying that demethylation of H3K4 by RBR-2 and SPR-5 contributes to the effect of insulin/IGF-1 signaling on longevity. Further, roles for the H3K4 demethylases *rbr-2* and *spr-5* were detected in germ cell maintenance over the generations under conditions of high temperature stress, which was exacerbated in strains where function of both genes was reduced. Thus, H3K4 demethylation may repress proliferative and postmitotic aging in response to stressful circumstances that include high environmental temperatures as well as genetic activation of a stress response pathway.

## Results

### Deficiency for *rbr-2* does not affect somatic morphology

**Figure 1:**
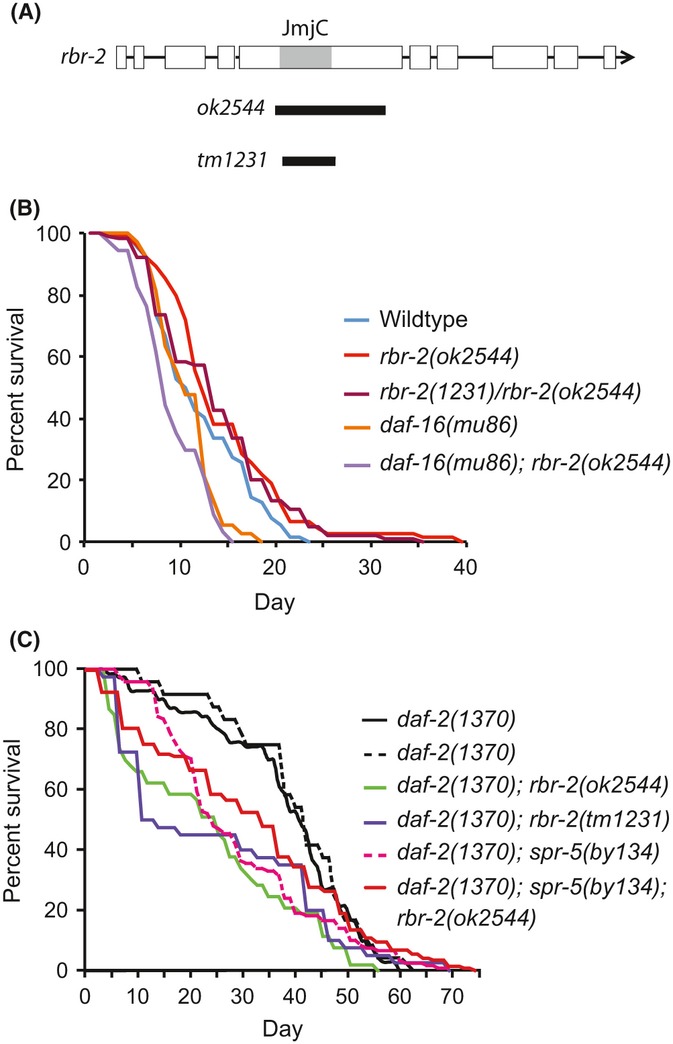
H3K4 demethylases and somatic longevity. (A) Gene structure of *rbr-2* deletion alleles *ok2544* and *tm1231*. The Jumanji C domain is shown in gray. Interaction of (B) *rbr-2(ok2544)* with *daf-16(mu16)* and (C) *daf-2(e1370)* with *rbr-2* or *spr-5* on lifespan. For (C), strains with solid lines were examined in the same experiment, while strains with a dotted line were examined together in a separate experiment. Mean lifespan and statistics are presented in Table S2.

In order to characterize *rbr-2*, we utilized two deletion alleles of *rbr-2*, *tm1231*, and *ok2544*. Both mutations cause in-frame deletions in exon 5 that remove the JmjC domain and therefore should lack demethylase activity (Fig. 1A). These deletions were verified by PCR genotyping and outcrossed four times in order to remove unlinked mutations that were generated during the creation of each deletion. Previous analysis of *rbr-2(tm1231)* revealed a high percentage of animals with vulval defects (Christensen *et al*., 2007). Moreover, RNAi knockdown of *rbr-2* in a background that displays a highly penetrant Multivulva phenotype at high temperature, *lin-15(n765ts)*, elicited Multivulva and Vulvaless phenotypes at low temperature, suggesting that the RBR-2 demethylase may interact with chromatin factors involved in the synthetic multivulva-B pathway (Ferguson & Horvitz, 1989; Lu & Horvitz, 1998; Solari & Ahringer, 2000; Christensen *et al*., 2007). In our hands, the initial lines of *rbr-2(tm1231)* exhibited weakly penetrant Protruding Vulva and Vulvaless phenotypes and continued to do so after being outcrossed. However, while initial *rbr-2(ok2544)* lines showed a weakly penetrant Vulvaless phenotype (observed in 29% of animals), outcrossed lines did not exhibit vulval defects. In addition, we observed that *tm1231* adults had a strong Small phenotype, whereas *ok2544* animals were normal in size (Table S1).

As the *tm1231* and *ok2544* deletions had different phenotypes, we examined whether the *rbr-2* deletion isolate *tm1231* had an unlinked mutation that elicits vulval or body size defects. We attempted to separate *rbr-2(tm1231)* from tightly linked mutations by singling Unc-non-Dpy recombinants from *+ rbr-2(tm1231) +/unc-24*+ *dpy-20* heterozygotes to create *unc-24 rbr-2 (tm1231)* double mutants. One *unc-24 rbr-2(tm1231)* strain was generated and continued to exhibit Vulvaless worms in 17% of the animals examined. However, *unc-24 rbr-2(tm1231) +*/*+ rbr-2(ok2544) dpy-20* transheterozygotes failed to display vulval morphology defects or a Small phenotype in adults (Table S1), both of which are easily observed for unoutcrossed *tm1231* homozygotes using a dissecting microscope. Because both deletions remove the JmjC domain of RBR-2, with *ok2544* being the larger deletion (Fig. 1A), deficiency for *rbr-2* demethylase activity is unlikely to perturb vulval morphology or body size.

### H3K4 demethylation promotes longevity of *daf-2* adults

It was previously reported that RNAi knockdown of *rbr-2* in wild-type adults results in an increase in lifespan at 25 °C (Lee *et al*., 2003; Ni *et al*., 2012). However, the *rbr-2(tm1231)* strain has been reported to display reduced longevity (Greer *et al*., 2010), and overexpression of a *GFP::rbr-2* fusion protein can extend lifespan of adult wild-type animals at 20 °C (Greer *et al*., 2010). At 20 °C, our outcrossed *rbr-2(ok2544)* strain exhibited both a longer mean and maximum lifespan (*P* < 0.05) (Table 1; Fig. S1). At 25 °C, both mean and maximum lifespan were also extended for *rbr-2(ok2544)* adults (*P* < 0.01), as well as for *unc-24 rbr-2(tm1231) +*/*+ rbr-2(ok2544) dpy-20* transheterozygotes (*P* < 0.01), in comparison with wild-type controls (Table 1; Fig. 1B). Log-rank analysis showed that these findings are significant, and two-way ANOVA showed no difference in the effect at two different temperatures (*P* < 0.678). Finally, *daf-16* single mutants were modestly short-lived in comparison with wild-type (*P* < 0.001), consistent with previous reports (Kenyon *et al*., 1993; Lakowski & Hekimi, 1996; Lin *et al*., 2001; Larsen *et al*., 2005), and *rbr-2* deficiency did not modify lifespan in a *daf-16* background (*P* < 0.1). Thus, the longevity of *rbr-2* single mutants may be mediated via DAF-16 (Fig. 1B).

**Table 1:** An *rbr-2(ok2544)* deficiency results in increased lifespan at 20 and 25 °C

Strains	Temperature (°C)	Mean lifespan‡	Maximum lifespan	No. death/censored (no. trial)
Wild-type	20	19.52 ± 1.03	33	33/2 (1)
*rbr-2(ok2544)*	20	22.25 ± 0.97*	37	64/11 (1)
Wild-type	25	12.94 ± 0.33	25	305/87 (3)
*rbr-2(ok2544)*	25	14.91 ± 0.73**	40	74/26 (1)
*rbr-2(1231)/rbr-2(ok2544)*	25	14.22 ± 0.61**	36	105/45 (2)
*daf-16(mu86)*	25	11.29 ± 0.21†	21	334/101 (3)
*daf-16(mu86)*; *rbr-2(ok2544)*	25	10.88 ± 0.41†	19	66/34 (2)

‡ Days ± SEM.

* *P* < 0.050 compared to the wild-type control using Mantel–Cox log-rank test.

** *P* < 0.010 compared to the wild-type control using Mantel–Cox log-rank test.

† *P* < 0.001 compared to the wild-type control using Mantel–Cox log-rank test.

Although *rbr-2* has not been identified in many studies examining downstream targets of DAF-2/DAF-16 signaling (Jones *et al*., 2001; McElwee *et al*., 2003; Murphy *et al*., 2003; Halaschek-Wiener *et al*., 2005), Lee *et al*. (2003) identified putative DAF-16 target sites genomewide, one of which corresponded to the *rbr-2* locus, and then rigorously showed that *rbr-2* was down-regulated in *daf-2* adults. We therefore assessed the lifespans of *daf-2(e1370)*; *rbr-2(ok2544)* and *daf-2(e1370)*; *rbr-2(tm1231)* double mutants. Sharp reductions in mean lifespan were observed for *daf-2(e1370)*; *rbr-2* double-mutant strains in comparison with long-lived *daf-2(e1370)* controls at 25 °C (*P* < 0.050 and *P* < 0.001 for *ok2544* and *tm1231*, respectively) (Fig. 1C; Table S2). Furthermore, in a *daf-2(e1368)* background, which is less long*-*lived at 25 °C, both *tm1231* and *ok2544* shortened median lifespan by 11.5% (*P* < 0.7) and 3.4% (*P* < 0.2), respectively (Table S2; Fig. S2). Our data imply that the RBR-2 H3K4 demethylase contributes to the extended lifespan of *daf-2* mutants and that the role of RBR-2 is most apparent for the pronounced longevity phenotype of *daf-2* allele *e1370*, which is commonly employed in studies of aging. Alternatively, it is also possible that a deleterious synthetic interaction between *daf-2* and H3K4 demethylases could lead to shortening of lifespan.

We next tested the SPR-5 H3K4 demethylase and found that *spr-5(by134)*; *daf-2(e1370)* double mutants had a significantly attenuated lifespan in comparison with *daf-2(e1370)* single mutants (*P* < 0.01) (Fig. 1C; Table S2). Furthermore, we tested for additivity between the deleterious effects of *spr-5* and *rbr-2*. *spr-5*; *daf-2(e1370)*; *rbr-2* triple mutants were short-lived in comparison with *daf-2(e1370)* single-mutant controls (Fig. 1C; Table S2) (*P* < 0.2), but were not shorter-lived than *spr-5*; *daf-2(e1370)* or *daf-2(e1370)*; *rbr-2* double mutants. Thus, RBR-2 and SPR-5 H3K4 demethylases were nonadditive with respect to their roles in promoting longevity of *daf-2* mutants, consistent with these two genes acting via the same mechanism, H3K4 demethylation, to silence loci that contribute to aging.

**Figure 2:**
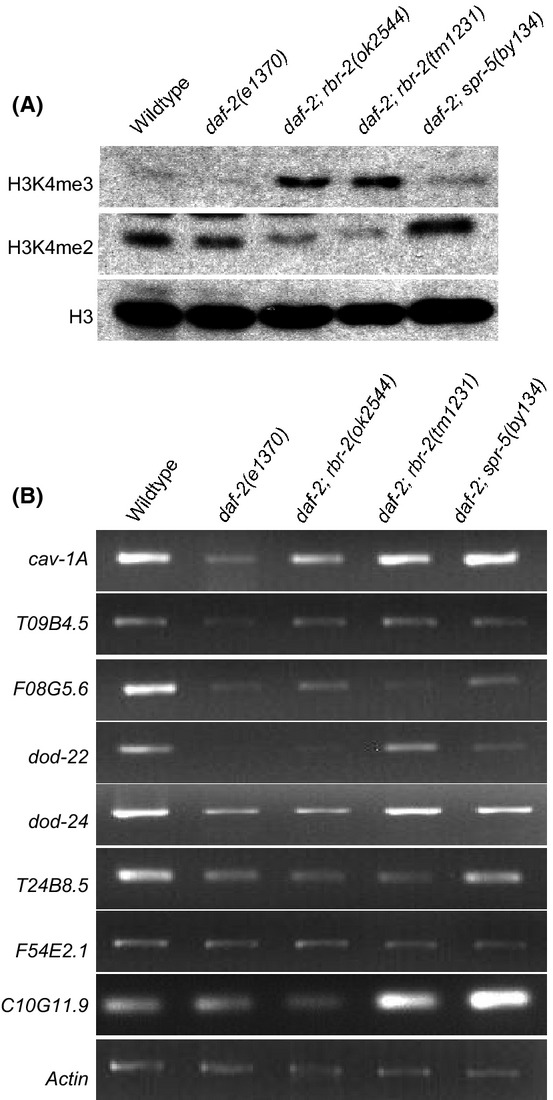
The effects of *rbr-2* and *spr-5* mutations on H3K4 methylation and on the expression of selected genes in a *daf-2(e1370)* background. (A) A western blot of protein extracts probed with antibodies against H3K4me3, H3K4me2, and H3. (B) Relative gene expression by semi-quantitative RT–PCR in the wild-type, *daf-2(e1370)* and *daf-2(e1370);rbr-2* and *spr-5*; *daf-2(e1370)* double mutants.

RBR-2 promotes H3K4me3 demethylation, whereas SPR-5 promotes H3K4me2 demethylation (Christensen *et al*., 2007). H3K4me3 levels were comparable in wild-type, *daf-2(e1370)* and *spr-5*; *daf-2* strains (Fig. 2A; Table S3). However, H3K4me3 levels were higher in *daf-2*; *rbr-2* double mutants (Fig. 2A; Table S3). Consistent with a role of SPR-5 in H3K4me2 demethylation, we found that H3K4me2 levels were higher in *spr-5*; *daf-2* in comparison with *daf-2* single-mutant controls, but lower in *daf-2*; *rbr-2* mutants (Fig. 2A; Table S4). Together, these results suggest that H3K4 demethylation may occur sequentially, where RBR-2 first acts on H3K4me3 and then SPR-5 demethylates H3K4me2.

To find targets of RBR-2 or SPR-5 in *daf-2* mutants, we examined a total of 17 genes previously shown to be silenced when *daf-2* is deficient, or de-silenced when *rbr-2* or *spr-5* is deficient (Murphy *et al*., 2003; Katz *et al*., 2009; Greer *et al*., 2010). RNA was harvested from wild-type, *daf-2* single mutants, and *spr-5*; *daf-2* or *rbr-2*; *daf-2* double mutants, and cDNA was made and normalized by RT–PCR using *act-1*. We found that six genes were repressed in *daf-2*, but not in wild-type RNA samples: *cav-1A*, *T09B4.5*, *F08G5.6*, *dod-22*, *dod-24* and *T24B8.5* (*n* = 2 RT–PCR experiments for two independently created cDNA aliquots per genotype) (Fig. 2B). *cav-1A* and *T09B4.5* were consistently up-regulated in *spr-5*; *daf-2* or *rbr-2*; *daf-2* double mutants (Fig. 2B). Expression of the remaining four genes was restored for only a subset of *spr-5*; *daf-2* or *rbr-2*; *daf-2* genotypes. In addition, we analyzed the twelve sperm genes that were previously shown to be repressed by SPR-5 (Katz *et al*., 2009). We confirmed that nine of these genes were desilenced in *daf-2*; *spr-5* mutants compared to *daf-2* alone, but did not show changes in *daf-2(e1370)* vs. wild-type as represented by *C10G11.9* (Fig. 2B). Further, many were also consistently elevated in *daf-2; rbr-2(tm1231)* double mutants, which was not observed for *daf-2; rbr-2(ok2544)* (Fig. 2B). Only one of the 12 sperm genes, *C02F5.5*, was repressed in *daf-2* compared to wild-type. C02F5.5 exhibited an expression profile similar to *T24B8.5* (Fig. 2B). Together, our data imply that a subset of genes that are silenced when *daf-2* is deficient may be targeted for silencing via RBR-2 and SPR-5 demethylases.

### Effects of *rbr-2* on fertility

**Figure 3:**
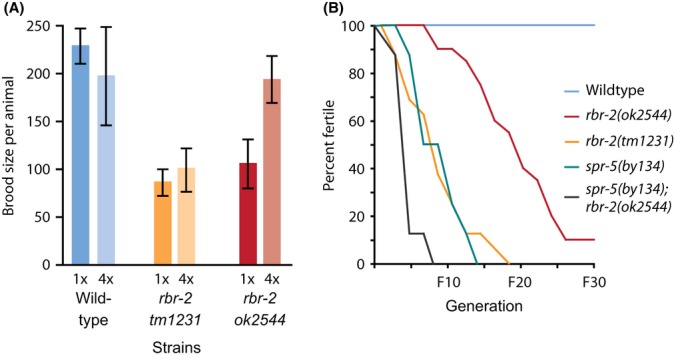
Deficiencies in H3K4 demethylation promote increased fertility defects at 25 °C. (A) Brood size at 20 °C for N2 wild-type, *rbr-2(tm1231)* and *rbr-2(ok2544)* after one (1X) or four (4X) outcrosses. (B) The effect of *rbr-2* and *spr-5* mutations on fertility until the F30 generation when propagated at 25 °C.

Deficiency for *spr-5* has been previously reported to result in high levels of sterility after growth for many generations at 20 °C (Katz *et al*., 2009). *rbr-2(ok2544)* and *rbr-2(tm1231)* strains that had been outcrossed a single time had lower brood sizes in comparison with wild-type controls (Fig. 3A), whereas four outcrosses led to wild-type levels of fertility for *ok2544*, but not for *tm1231* (Fig. 3A). Thus, the effects of *tm1231* on brood size are independent of the histone demethylase activity of *rbr-2*.

Transgenerational effects of *rbr-2* deficiency on fertility were studied by propagating strains for many generations at 20 °C, or at the higher stressful temperature of 25 °C, using the mortal germline assay, in which small population bottlenecks of 6 L1 larvae are transferred every two generations (Smelick & Ahmed, 2005). In early generations, as well as after many generations of growth at 20 °C, outcrossed *rbr-2* strains displayed very few or no sterile animals when assessed either by singling cohorts of 40 L4 larvae or by scanning large populations of adults for sterile animals with a germline proliferation (Glp) defect (sterile adult animals displaying an empty uterus that is clearly visible under a dissecting microscope). However, growth at 25 °C resulted in immediate drops in fertility for both *ok2544* and *tm1231*. Subsequent propagation at 25 °C resulted in large numbers of Glp animals for both alleles, ultimately leading to complete sterility for all *rbr-2 tm1231* lines by generation 16 (*n* = 17 lines). In contrast, only 90% of *rbr-2(ok2544)* lines became sterile during 30 generations of growth at 25 °C (*n* = 20 lines) (Fig. 3B). Thus, deficiency for *rbr-2* causes a Mortal Germline (Mrt) phenotype that is incompletely penetrant after 30 generations of growth (Ahmed & Hodgkin, 2000).

**Figure 4:**
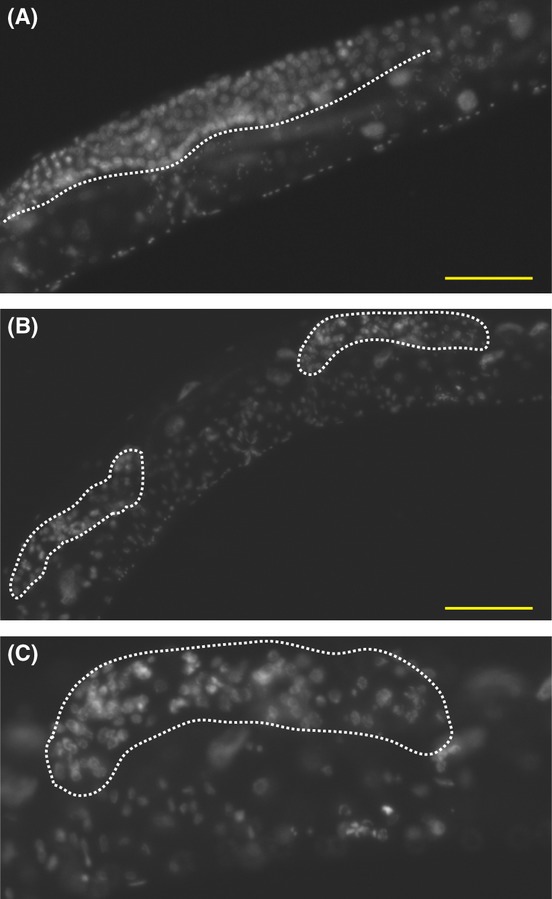
Late-generation *rbr-2* transheterozygotes display atrophied germlines. (A) Wild-type germline of *unc-24 rbr-2(1231)+/+ rbr-2(ok2544) dpy 24* F3 generation grown at 25 °C. (B) Atrophied germlines found in sterile late-generation *unc-24 rbr-2(1231) +/+ rbr-2(ok2544) dpy-24* adults from independently propagated lines. (C) Magnified image of germline arm in (B). Dotted lines outline the germline. Bar represents 100 μm.

The morphology of sterile adult hermaphrodite germlines of *tm1231* and *ok2544* homozygotes as well as *unc-24 rbr-2(tm1231) +*/*+ rbr-2(ok2544) dpy-20* transheterozygotes was visualized by fluorescence microscopy using the DNA-intercalating dye 4′,6-diamidino-2-phenylindole (DAPI). Fertile *rbr-2* transheterozygotes in early generations at 25 °C had wild-type-sized germlines (Fig. 4A), whereas late-generation sterile adults often exhibited small or empty germline arms (Fig. 4B,C). Consistently, sterile late-generation *rbr-2(tm1231)* adults exhibited empty or small germlines (42%, *n* = 128), but also displayed a germline overproliferation phenotype not seen in the transheterozygotes in late generations (Table 2). High-resolution analysis of the number of DAPI spots in oocytes arrested in diakinesis revealed 5.69 ± 0.1 for wild-type controls, 5.74 ± 0.1 for sterile *rbr-2(tm1231)* adults, and 5.89 ± 0.1 for sterile transheterozygotes (Table 2), indicating that sterility is not caused by chromosome fusions, as occurs in *C. elegans* telomerase mutants (Meier *et al*., 2006).

**Table 2:** Phenotypes of sterile adults deficient for H3K4 demethylases

Strains	Wild-type (%)	Atrophy† or empty‡ (%)	Prolif§ (%)	Other¶ (%)	Number of bivalents**
Wild-type	100	0	0	0	5.7 ± 0.1
*rbr-2(tm1231)*	40.3	44.2	13.2	2.3	5.7 ± 0.1
*rbr-2(ok2544)*	46.5	41.1	13.4	0	5.7 ± 0.1
*rbr-2(tm1231)/rbr-2(ok2544)*	33.3	57.8	0	8.9	5.9 ± 0.1
*spr-5(by134)*; *rbr-2(ok2544)*	35.5	41.9	6.5	16.1	5.8 ± 0.2
*spr-5(by134)*	36.7	61.2	2	0	5.4 ± 0.1*

† Less than half the number of germ cells as compared to wild-type.

‡ No germ cells.

§ Overproliferated germline.

¶ Unusual germline morphology including fragmented chromosomes and endomitotic oocytes.

** Mean ± SEM.

* *P* < 0.05 compared to wild-type.

### A second H3K4 demethylase represses proliferative aging of germ cells at high temperature

The *spr-5* (KDM1A) H3K4 demethylase allele *by101* has been shown to cause high levels of sterility (~90%) when propagated at 20 °C for many generations (Katz *et al*., 2009), which was enhanced by the loss of *amx-1* (KDM1B). However, loss of function of all *C. elegans* KDM1 genes did not prevent H3K4 demethylation in the germline precursor cells Z2 and Z3 in early generations. We therefore hypothesized that KDM1 fertility defects of *spr-5* mutants might be exacerbated by deficiency for the KDM5 demethylase *rbr-2*. *spr-5* alleles were isolated based on their ability to suppress the egg-laying defects seen in *sel-12* presenilin mutants (Eimer *et al*., 2002; Jarriault & Greenwald, 2002). We did not use the *spr-5* allele *by101* because it was the first *spr-5* allele isolated and was the longest cultured of the *spr-5* alleles and because *spr-5* strains have recently been shown to be deficient for meiotic double-strand break repair (Nottke *et al*., 2011). Thus, *by101* may have a significant load of spontaneous background mutations due to extended culturing (Eimer *et al*., 2002; Jarriault & Greenwald, 2002). Furthermore, *by101* is caused by a *Tc3* transposon insertion and exhibits two *spr-5* transcripts: one that is longer than the wild-type, presumably containing the *Tc3* insertion, and a second transcript of approximately wild-type length that likely results from transposon excision (Eimer *et al*., 2002). Instability of the *by101 Tc3* transposon insertion, possibly due to alterations in epigenetic marks or to novel mutations in the *by101* strain, could have contributed to the fertility phenotypes previously reported for this allele (Katz *et al*., 2009). Because all *spr-5* alleles have a very similar strong suppressor of *sel-12* phenotype, we chose to examine the *by134* allele, which has an early stop codon that severely reduces transcript levels and truncates the SPR-5 protein (Eimer *et al*., 2002). Thus, *by134* is likely to be a very strong loss of function, or null, allele of *spr-5* and should have no enzymatic activity (Eimer *et al*., 2002).

We observed a small number of Glp animals in outcrossed *spr-5(by134)* strains that had been grown for over 60 generations at 20 °C, with 1 of 49 singled L4s exhibiting no progeny. Brood size counts of non-Glp animals revealed lower than wild-type brood sizes of 79.3 ± 8.22 progeny per worm (*n* = 27). However, passaging 6 L1s at 20 °C every week failed to generate a Mortal Germline phenotype (*n* = 4 strains), even after propagation for over 50 generations (Fig. S3). We note, however, that our preliminary observations during the initial isolation and study of *spr-5* mutations revealed that *spr-5(-)*; *sel-12(ar171)* strains exhibited reduced fertility and some sterility after 50 generations of continuous passaging. As previously reported (Katz *et al*., 2009), outcrossing these *spr-5* strains a single time rescued these defects. Thus, although epigenetic changes can accrue slowly and eventually limit fertility of *spr-5*; *sel-12* strains, and of *spr-5(by101)* single mutants (Katz *et al*., 2009), we conclude that null *spr-5* alleles can be maintained for many generations without severe reductions in fertility at 20 °C.

Because RBR-2 and SPR-5 demethylases could redundantly target H3K4Me2 methyl marks, we anticipated that deficiency for both proteins might result in a strong synthetic interaction. *rbr-2(ok2544)* hermaphrodites were crossed with *spr-5(by134)* males to create *spr-5*; *rbr-2* double mutants. Five *spr-5*; *rbr-2* double-mutant lines were propagated for more than 30 generations at 20 °C, but were not obviously different from wild-type controls and lacked Glp adults (Fig. S3). Further, levels of embryonic lethality and numbers of progeny were not significantly different from single-mutant or wild-type controls. However, ~65% of the *spr-5*; *rbr-2* double-mutant animals exhibited an egg-laying defect when they were 3-or 4-day-old adults, resulting in matricide, suggesting that at 20 °C, somatic rather than germ cell development is redundantly affected by these H3K4 demethylases.

Deficiency for *rbr-2* at high temperature elicited a late-onset Mrt phenotype (sterility after growth for many generations). *spr-5* strains all became sterile by generation 14 at 25 °C (*n* = 17/17 lines tested) (Fig. 3B). This Mrt phenotype was more rapid and penetrant than for *rbr-2* at 25 °C or for *spr-5* mutations at 20 °C (Katz *et al*., 2009). Moreover, four independently derived *spr-5(by134)*; *rbr-2(ok2544)* double-mutant strains became sterile by generation F8, indicating that deficiency for *rbr-2* enhances the Mrt phenotype of *spr-5* at 25 °C (*P* < 0.05; *n* = 19 lines in total, at least 2 lines tested per initial strain in addition to ten outcrossed lines) (Fig. 3B).

Analysis of sterile *spr-5* or *spr-5*; *rbr-2* adults by microscopy revealed phenotypes similar to those of sterile *rbr-2* mutants. For example, 5.76 ± 0.2 bivalents were observed for *spr-5*; *rbr-2* germlines that did contain oocytes (Table 2). Additional phenotypes were occasionally observed for *spr-5(by134)*, including disorganized DAPI-stained chromosomes in oocytes at the germline bend (Fig. S4A), endomitotic oocytes (Iwasaki *et al*., 1996), as well as a modest reduction in the number of bivalents per oocyte (5.4 ± 0.1; *P* < 0.05) (Fig. S4B–C).

### RBR-2 does not promote longevity of quiescent germ cells

**Figure 5:**
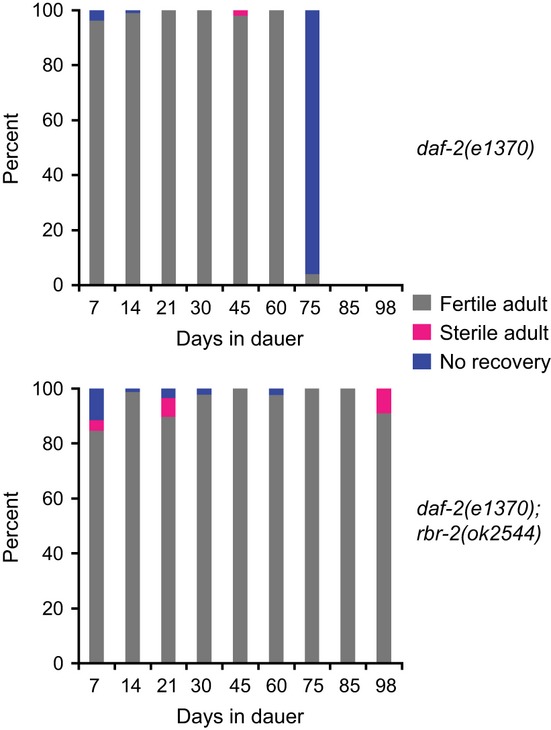
Quiescent germ cells do not require *rbr-2* for fertility. Recovery from the dauer stage for *daf-2(e1370)* and *daf-2(e1370)*; *rbr-2(ok2544)* dauers of different ages. Adults were considered fertile if any progeny were produced.

*rbr-2* deficiency results in a partially penetrant Mortal Germline phenotype at 25 °C, which suggests transmission and accumulation of a heritable form of damage, or ‘proliferative aging’, of germ cells over multiple generations of growth. We tested for an analogous role of *rbr-2* in the maintenance of quiescent germ cells over long periods of time using dauer larvae, a stress-induced larval stage that is long-lived. *daf-2* mutants arrest development as dauer larvae at 25 °C but can resume development and produce progeny when transferred to 15 °C. Cohorts of dauer larvae for *daf-2(e1370)*; *rbr-2(ok2544)* double mutants as well as *daf-2(e1370)* single-mutant controls were maintained at 25 °C for several months. Every 10–15 days, pools of dauers were singled and transferred to 15 °C for recovery. Both strains showed comparable levels of high viability, development to adulthood, and fertility for 60 days (Fig. 5). At 75 days, although *daf-2(e1370)* dauer larvae appeared paralyzed, they moved slightly when singled and showed movement on the recovery plate. However, they failed to recover at 15 °C. Unexpectedly, a high percentage of *daf-2(e1370)*; *rbr- 2(ok2544)* double-mutant larvae recovered and continued to display high levels of fertility when transferred to 15 °C until day 98, at which time the experiment was terminated. In contrast, most *rbr-2(ok2544)* strains that had been propagated continuously at 25 °C for 98 days (28 generations) became sterile (Fig. 3B). Thus, deficiency for *rbr-2* failed to compromise the maintenance of quiescent germ cells in dauer larvae. The prospect that *rbr-2* function could be detrimental to longevity of *daf-2*-mutant dauer larvae deserves further investigation, as most studies of the effects of *daf-2* on longevity concern adults. We speculate that regulation of gene expression by RBR-2 during dauer entry or dauer diapause could promote an optimal life history strategy that results in a trade-off in dauer longevity.

To further investigate the interaction of H3K4 demethylases with reduced insulin/IGF-1 signaling, we examined a role for *rbr-2*, as well as *spr-5*, in constitutive dauer formation of *daf-2(e1370)* mutants at 25 °C. At 25 °C, a strong dauer formation phenotype was observed for *daf-2(e1370)*, with no animals developing into adults, which was also seen for *spr-5*; *daf-2* and *daf-2*; *rbr-2* double mutants (Table S5). Thus, the defect in lifespan is not accompanied by a defect in dauer formation per se. At 20 °C, only a proportion of *daf-2(e1370)* larvae arrest as dauers, and no consistent effect was observed for both *rbr-2* alleles (plenty of dauers occurred in both cases) (Table S5). However, *spr-5(by134)*; *daf-2* double mutants exhibited few dauers at 20 °C (Tables S5 and S6), suggesting that *spr-5* promotes dauer formation in conditions where dauer formation is weakly induced. Thus, while both *rbr-2* and *spr-5* promote longevity in *daf-2* mutant adults, only *spr-5* promotes dauer formation in *daf-2* mutants, and *rbr-2* promotes dauer aging. This reveals selective roles for *rbr-2* and *spr-5* on different aspects of diapause and longevity in IIS mutants.

## Discussion

Longevity assays in model invertebrates typically focus on adult somatic cells that are quiescent (*C. elegans*) or that only possess small populations of stem cells (*Drosophila*). Studies of proliferative aging in *C. elegans* have defined a number of mutants that are deficient for telomerase-mediated telomere replication. However, neither telomere length nor telomerase affects postmitotic aging of *C. elegans* adults (Meier *et al*., 2006). In this study, we define two proteins that can repress aging in both postmitotic somatic cells and proliferating germ cells, RBR-2 and SPR-5. Therefore, these two forms of aging may be related, and histone demethylase activities could be broadly relevant to the regulation of longevity in diverse organisms (Misteli, 2010).

It has been suggested that RBR-2 has anti-aging functions, as *rbr-2(tm1231)* adults were short-lived (Greer *et al*., 2010). However, it was also reported that RNAi knockdown of *rbr-2* causes increased longevity of the enhanced RNAi strain *rrf-3* (Lee *et al*., 2003) and of the N2 wild-type strain at both 20 and 25 °C (Ni *et al*., 2012). Consistently, our analysis of *rbr-2(ok2544)* revealed a significant, if modest, increase in mean lifespan. *rbr-2(ok2544)* failed to complement *rbr-2(tm1231)* for this increased longevity phenotype. Thus, the *tm1231* allele is not neomorphic but has unusual phenotypes that could be due to additional closely linked mutations in this genetic background; such issues of genetic background have previously confounded studies of the biology of aging in *Drosophila* and *C. elegans* (Burnett *et al*., 2011; Viswanathan & Guarente, 2011). The differences between the *tm1231* and *ok2544* alleles are unlikely due to residual demethylase activity as both alleles delete the conserved JmjC domain (Fig. 1A). However, we cannot rule out the possibility that the *tm1231* allele could have unique and possibly interesting properties that are not due to linked mutations, such as a direct effect on lifespan that suppresses the anti-aging effects caused by *ash-2* RNAi (Greer *et al*., 2010). The *tm1231* deletion is predicted to result in a protein product that retains some RBR-2 sequences, including a C_5_HC_2_ zinc finger domain, which are absent from the predicted protein product of *ok2544*. We conclude that *rbr-2(ok2544)* may be a better mutation for studying null RBR-2 function than *rbr-2(tm1231)*.

We found that members of two classes of H3K4 demethylase are required for extended longevity in *daf-2(e1370*) adults. This suggests an anti-aging function for RBR-2, consistent with the observation that overexpression of *rbr-2* in germ cells extends lifespan of otherwise wild-type adults (Greer *et al*., 2010). The interaction between *rbr-2* and *daf-2* in *C. elegans* may be conserved in mammals, as IGF-1 receptor and RBP-2 demethylase activities were shown to mediate the emergence and maintenance of a ‘drug-tolerant’ state of cancer cell lines (Sharma *et al*., 2010). A recent report showed that the UTX-1 H3K27 demethylase and the T26A5.5 H3K36 demethylase are pro-aging and that deficiency for *utx-1* extends lifespan in a manner that mimics reduced levels of *daf-2* signaling (Maures *et al*., 2011), which may be due to the regulation of *daf-2* gene expression (Jin *et al*., 2011). We speculate that removal of H3K27 silencing marks by UTX-1 could promote aging in a manner that resembles the anti-aging effects of RBR-2 and SPR-5, whose H3K4 demethylase activities may promote silencing of pro-aging genomic loci, such as *cav-1A* and *T09B4.5*, in the context of *daf-2* deficiency (Figs 1C and 2). *daf-2*-dependent epigenetic modifications could be relevant to the activity of the DAF-16/Foxo transcription factor, or those of its targets.

Although both *rbr-2* deletions elicit a Mortal Germline phenotype at a higher temperature, the partially penetrant Mortal Germline phenotype of *ok2544* is likely to reflect the true role of RBR-2 in repression of transgenerational aging in the germline. In addition, defects in either *rbr-2* or *spr-5* only affected germ cells that were continuously cycling at higher temperatures, as quiescent germ cells from dauer larvae did not exhibit strong fertility defects during an analogous length of time (Fig. 2). Thus, cell proliferation is likely required for the accumulation of transgenerational stress in *rbr-2* and *spr-5* mutants. An alternative explanation is that pathways that establish quiescence and longevity in dauer larvae (Narbonne & Roy, 2006) could repress the stress that is caused by deficiency for *rbr-2* or *spr-5* in germ cells.

A previous study showed that KDM1 enzymes, especially SPR-5, affect germline maintenance over many generations at 20 °C (Katz *et al*., 2009). However, we found that the severe *spr-5* mutation *by134* resulted in modest effects on fertility at 20 °C and that *spr-5* mutants can be maintained for many generations, consistent with a recent report from the laboratory of M. Colaiacovo (Nottke *et al*., 2011). We suggest that the variable effects of *spr-5* on germline maintenance may be allele specific, but could also be explained by factors that affect the epigenetic landscape of *spr-5* strains, such as laboratory growth conditions or genetic backgrounds that were employed to perform crosses. One clear difference in the way we conducted experiments from those reported by Katz *et al*. (2009) is that we maintained strains in a wild-type (N2) background as much as possible. Double-mutant strains were constructed by directly crossing single-mutant strains or by using a limited number of genetic markers. Katz *et al*. maintained *spr-5(by101)* strains in a heterozygous state, balanced over *hT2*, a marked reciprocal translocation, prior to starting experiments. The effects of using the *hT2* balancer, which has been subjected to several rounds of mutagenesis, on quantitative aspects of aging and fertility over many generations are unknown.

In our hands, much stronger effects on fertility were observed for *rbr-2* and *spr-5* mutants at 25 °C. Mutations in these genes are not known to be temperature sensitive, suggesting that they uncover a temperature-sensitive requirement for H3K4 demethylase activity. Due to increased molecular motion at higher temperatures, chromatin may become more open to transcription, leading to a greater requirement for proteins that facilitate transcriptional repression such as H3K4 demethylases. Alternatively, high temperature might induce the expression of stress-related genes, such as heat-shock proteins, and expression of this stress program might be tempered by the action of genes that repress transcription. A recent study has shown that the *bn129* allele of the H3K4 histone methyltransferase *set-2* (KMT2) has a temperature-sensitive Mrt phenotype (Xiao *et al*., 2011). In addition, deficiencies in synMuvB proteins elicit a larval arrest phenotype caused by failure to remodel the germline chromatin program at higher temperature (Petrella *et al*., 2011). Thus, high temperature may be a sensitized condition capable of detecting biological functions of chromatin-modifying proteins, such as facilitating the dynamics and/or balance of H3K4 methylation in the maintenance of fertility.

Although strains deficient for both demethylases did not exhibit strong fertility defects at 20 °C, the Mrt phenotype of *spr-5*; *rbr-2* double mutants at 25 °C was significantly faster than that of either single mutant. It is possible that these demethylases interact with common as well as distinct genomic loci to repress transgenerational aging. Despite being able to demethylate both Me3 and Me2 from H3K4, RBR-2 plays a less important role in repressing transgenerational stress at 25 °C than SPR-5, which demethylates Me2 and Me1 (Christensen *et al*., 2007; Katz *et al*., 2009). SPR-5 may be more important than RBR-2 for promoting germ cell immortality because RBR-2 has only weak activity in removing Me2 and is more efficient at removing Me3 (Fig. 2A) (Christensen *et al*., 2007). Alternatively, SPR-5 may have a stronger phenotype because KDM1A enzymes can also demethylate H3K9 (Metzger *et al*., 2005) and other nonhistone proteins (Nicholson & Chen, 2009). Given their overlapping mutant phenotypes and the reduced levels of H3K4me2 in *daf-2*; *rbr-2* mutants (Fig. 2), we propose that RBR-2 and SPR-5 may function sequentially on distinct H3K4 methyl marks of common histone targets to repress proliferative or postmitotic aging.

Our findings that RBR-2 and SPR-5 impact cell proliferation and aging in *C. elegans* could be relevant to human homologs of these proteins, which have been implicated in the regulation of stem cell fate and in cancer biology. For example, in embryonic stem cells, LSD1 (the human homolog of SPR-5) helps to maintain the balance between differentiation and self-renewal (Adamo *et al*., 2011). LSD1 has also been implicated in many cancers, and selective inhibitors of LSD1 are being investigated as possible anticancer agents (Wang *et al*., 2011). RBP-2, the human homolog of RBR-2, is released from promoters of genes that are expressed during cellular differentiation (Christensen *et al*., 2007), consistent with a role for heterochromatin in maintaining a pluripotent state. In addition, RBP-2 promotes cancer cell survival and proliferation (Roesch *et al*., 2010; Zeng *et al*., 2010; Blair *et al*., 2011). Given that the anti-aging roles of RBR-2 and SPR-5 in both proliferative and postmitotic aging occur under conditions of temperature stress or a *daf-2* genetic background that may mimic stressful conditions, we speculate that a conserved function of H3K4 demethylases may be to repress cellular aging by modifying chromatin in response to physiological stress.

## Experimental procedures

### Strains

Unless noted otherwise, all strains were cultured at 20 °C on nematode growth medium (NGM) plates seeded with *Escherichia coli* OP50. Mutations used include *dpy-24(s71) I*, *daf-16(mg50) I*, *daf-16(mu86) I*, *spr-5(by134) I*, *glp-4(bn2) I*, *daf-2(e1368) III*, *daf-2(e1370) III*, *unc-24(e120) IV*, *rbr-2(tm1231) IV*, *rbr-2(ok2544) IV*, *dpy-20(e1282) IV*.

*rbr-2* mutations were outcrossed vs. an *unc-24 dpy-20* marker strain that itself had been outcrossed three times vs. N2 wild-type. Freshly isolated homozygous *rbr-2* F2 lines were established, and nonstarved strains were used for the analysis of germ cell immortality. For longevity experiments, *rbr-2* stocks that had been starved for varying periods of time after outcrossing were used. *spr-5(by134)* was outcrossed once vs. *glp-4(bn2)* and confirmed to be homozygous by DNA sequencing. Generating the double-mutant strain is found in supplemental methods.

### Western blot

Worm extracts were made from the indicated *daf-*2 and wild-type strains by shifting 200 L4s from 15 to 25 °C and allowing them to grow for 2 days. Worms were then collected and resuspended in 2× Laemmli buffer containing 5% beta-mercaptoethanol. Samples were boiled for 10 min, and proteins were separated in a 15% SDS-PAGE gel. The gel was transferred to nitrocellulose and blotted for H3 (abcam #ab1791) to make sure samples were normalized. The blot was then stripped and probed for H3K4me3 (abcam #ab8580) as well as H3K4me2 (Millpore #07-030). Quantification was performed using ImageJ software (US National Institutes of Health, Bethesda, MD, USA).

## Supporting Information

**Fig. S1** *rbr-2(ok2544)* affects longevity at higher temperatures.

**Fig. S2** The effects of *rbr-2* on *daf-2(e1368)*-mediated longevity.

**Fig. S3** Mutations in H3K4 demethylases do not affect fertility for strains propagated at 20 °C.

**Fig. S4** DAPI-stained oocytes with chromosome defects.

**Data S1** Methods.

**Table S1** Smaller body size and vulval defects in *rbr-2(tm1231)* but not *rbr-2(ok2544)*. **Table S2** Deficiencies for H3K4 demethylase activity decreases longevity in a *daf-2(e1370)* background. **Table S3** Dauer formation and L1 or L2 larval arrests of progeny from *daf-2(e1370)* and *daf-2(e1370)* H3K4 demethylase double mutants that were shifted from 15 °C to either 20 or 25 °C as L4 larvae. **Table S4** Dauer formation and L1 or L2 larval arrests of progeny from *daf-2(e1370)*, H3K4 demethylase mutants that were shifted from 15 °C to either 20 or 25 °C as embryos. **Table S5** Quantification of H3K4me2 compared to total H3K4 levels. **Table S6** Quantification of H3K4me3 compared to total H3K4 levels.
