# Supplementary material for: H3K4 demethylase activities repress proliferative and postmitotic aging
Source: Aging Cell. 2013 Nov 19;13(2):245–53. doi: 10.1111/acel.12166 (PMC4020274; doi:10.1111/acel.12166)
Supplement: Supplementary file 5 — Data S1 Methods. [file acel0013-0245-sd5.doc]

**Data S1**

**Supplemental Methods**

**Generation of double mutant strains**

To generate *daf-2; rbr-2(ok2544)* double mutants, *daf-2; unc-24* strains were made and *daf-2/+; unc-24 / +* heterozygous males were crossed with *rbr-2(ok2544)* hermaphrodites, non-Unc F2 dauers were selected from F1 plates exhibiting Dauer and Unc progeny at 25°C, followed by Dauer recovery at 15°C and selection against *unc-24*. To generate *daf-16; rbr-2(ok2544)* double mutants, *dpy-24(s71); rbr-2(ok2544)* and *daf-16; unc-24(e120)* strains were created. *rbr-2(ok2544)* males were then crossed with *dpy-24(s71); rbr-2(ok2544)* to generate *dpy-24(s71) / +; rbr-2(ok2544) / rbr-2(ok2544)* males, which were crossed with *daf-16; unc-24(e120)* hermaphrodites, and F1 with both Dpy and Unc F2 progeny were used to create and *daf-16;rbr-2(ok2544)* double mutants by selecting against Dpy and Unc phenotypes.

The *spr-5(by134); rbr-2(ok2544)* double mutants were generated by direct crosses and sequencing of many F2 progeny. The F3 progeny of 80 F2 hermaphrodites from this cross were scored for embryonic lethality, sterility, a reduced brood size, or slow growth at 20ºC and then genotyped for the *rbr-2* and *spr-5* mutations. A phenotype was observed for only two F2 animals, which produced ~10% Glp F3 progeny, and these were *spr-5 +/-; rbr-2 +/-* heterozygotes. Five of the remaining 78 lines, which is very close to the expected 1/16^th^, were *spr-5; rbr-2* double mutants that appeared wildtype in the F3 generation. However, we initially attempted to create this genotype using visible markers by crossing *glp-4(bn2); unc-24(e120)* with *spr-5(by134)* or *rbr-2(ok2544)* males, but progeny from these crosses did not segregate into the expected phenotypes and yielded abnormal animals that were sterile. This result highlights the effects that background mutations can have on phenotypes of *spr-5* and *rbr-2* strains.

**Analysis of germline mortality**

Mortal Germline assays were performed by transferring 6 L1 larvae to fresh NGM plates once a week (every two generations), as previously described (Smelick and Ahmed, 2005).

**Analysis of lifespan**

Adult lifespans were performed using 6 animals per NGM plate and initiated with non-starved L4 stage animals (day 0). Young adults were transferred to fresh plates daily until egg-laying ceased, and then transferred weekly. Animals that were lost or bagged were censored and longevity analysis was performed using the Mantel-Cox log-rank test. Significance of mean was calculated using a Student’s T-test. Results were confirmed using the Online Application for Survival Analysis (OASIS) website (Yang et al., 2011).

**DAPI staining**

In order to examine germlines, L4 animals from indicated strains were plated onto freshly seeded plates and allowed to grow to young adults overnight. Animals were then collected and stained with 4’,6-diamidino-2-phenylindole (DAPI) (Molecular Probes, Eugene, OR) as described (Ahmed and Hodgkin, 2000).

**RT-PCR**

Worms grown at 15°C were shifted to 25°C as L4s and continued to grow at 25°C for two days. Worms were collected and RNA was purified using TRIzol reagent (Ambion). cDNA was made from RNA using Superscript III (Invitrogen) according to the manufacturer’s instructions. cDNA was then normalized using the following primers for *actin-1*: GATATGGAGAAGATCTGGCATCA and GGGCAAGAGCGGTGATT. Primers for genes listed in Figure 2 are CTCGAGAACAACCATGCC and GACGCATGGAGCAGTAGTT for *cav-1A*, AAGTGACCACACCAAGCAG and CTTGGCATACGACCAATC for *T09B4.5*, ATAATGCGACTATTCCAGCG and AGCCGGAGTATGCGATG for *F08G5.6*, ATTGGCTACTCTGGTGTTACTGAAG and CCTACCGTTGGAGAGCCAT for *dod-22* , ATCAACATCGGGACGGAC and CATTCACCTGGAGTGGCA for *dod-24,* GTGCTTGTAGCTTACACTGCTTC and CATTGGCTGTGCAGTTGTA for *T24B8.5* , TTGCTCAACAAGTTATCCCAC and CAAGGGTAACTGCGAACG for *F54E2.1* and GTCTCCCGTGGATCGAAG and gaacgagagcgtgatgaaga for *C10G11.9* Results were confirmed twice and on two independently derived RNA sample sets.

**Detecting defects in dauer entry**

L4 animals with the indicated genotypes were allowed to lay eggs at either 20°C or 25°C for Table S5 and 15°C for Table S6. Progeny were then scored for arrest at L1, L2, and dauer. Those animals that did not arrest at these stages became adults and were included in the total score. Average percents for each of these stages was calculated and significance was analyzed using a Student’s T-test.

Supplemental Reference

Yang JS, Nam HJ, Seo M, Han SK, Choi Y, Nam HG, Lee SJ, Kim S (2011). OASIS: online application for the survival analysis of lifespan assays performed in aging research. *PLoS One* **6**, e23525.
