# Supplementary material for: H3K4 demethylase activities repress proliferative and postmitotic aging
Source: Aging Cell. 2013 Nov 19;13(2):245–53. doi: 10.1111/acel.12166 (PMC4020274; doi:10.1111/acel.12166)
Supplement: Supplementary file 6 — Table S1 Smaller body size and vulval defects in rbr-2(tm1231) but not rbr-2(ok2544). Table S2 Deficiencies for H3K4 demethylase activity decreases longevity in a daf-2(e1370) background. Table S3 Dauer formation and L1 or L2 larval arrests of progeny from daf-2(e1370) and daf-2(e1370) H3K4 demethylase double mutants that were shifted from 15 °C to either 20 or 25 °C as L4 larvae. Table S4 Dauer formation and L1 or L2 larval arrests of progeny from daf-2(e1370), H3K4 demethylase mutants that were shifted from 15 °C to either 20 or 25 °C as embryos. Table S5 Quantification of H3K4me2 compared to total H3K4 levels. Table S6 Quantification of H3K4me3 compared to total H3K4 levels. [file acel0013-0245-sd6.doc]

**Table S1. Smaller body size and vulval defects in *rbr-2(tm1231)* but not *rbr-2(ok2544)***

| Strain | Body Length ^a^ (no. of worms) | Vulval Defects |
| --- | --- | --- |
| wildtype | 1.12 +/- 0.01 (30) | none |
| *rbr-2(ok2544)* outcrossed | 1.10 +/- 0.01 (30) | none |
| *rbr-2(tm1231)* not outcrossed | 0.81 +/- 0.01 (30) | 30% Vulvaless |
| *rbr-2(tm1231)* outcrossed | 0.83 +/- 0.05 (30) | 27% Vulvaless |
| *rbr-2(tm1231)/ rbr-2(ok2544)* | 0.98 +/- 0.02 (32) | none |

^a^ mean body length in mm +/- SEM

**Table S2. Deficiency for H3K4 demethylase activity decreases longevity in a *daf-2(e1370)* background**

| Strain | Mean Lifespan^a^ | Maximum Lifespan (days) | No. death/censored (no. trial) |
| --- | --- | --- | --- |
| *daf-2(e1370)* | 38.4 +/- 1.5 | 59 | 172/40 (2) |
| *daf-2(e1370); rbr-2(ok2544)* | 25.2 +/- 2.1^b^ | 55 | 53/12 (1) |
| *daf-2(e1370); rbr-2(tm1231)* | 30.6 +/- 2.3^c^ | 57 | 40/39 (2) |
| *daf-2(e1370); spr-5(by134)* | 28.8 +/- 1.3^d^ | 70 | 121/9 (2) |
| *daf-2(e1370); spr-5(by134); rbr-2(ok2544)* | 32.9 +/- 1.6^e^ | 76 | 148/2 (1) |
| *daf-2(e1368)* | 23.4 +/- 0.9 | 36 | 84/32 (2) |
| *daf-2(e1368); rbr-2(ok2544)* | 22.6 +/- 1.7^e^ | 42 | 43/24 (1) |
| *daf-2(e1368); rbr-2(tm1231)* | 20.7 +/- 1.1^e^ | 33 | 63/32 (1) |

^a^ days ± SEM; ^b^ *P*≤0.001; ^c^ *P*<0.05; ^d^ *P*<0.01; ^e^ not significant

**Table S3. Quantification of H3K4me3 compared to total H3K4 levels.**

| Strains | ratio replicate 1  H3K4me3/H3K4 | ratio replicate (in paper)  H3K4me3/H3K4 |
| --- | --- | --- |
| | N2 H3K4me/ N2 H3K4 | | --- | | 0.323 | **0.055** |
| *daf-2(e1370)* H3K4me3/  *daf-2(e1370)* H3K4 | 0.225 | **0.029** |
| *daf-2(e1370);rbr-2(ok2544)* H3K4me3/  *daf-2(e1370);rbr-2(ok2544)* H3K4 | 0.381 | **0.221** |
| *daf-2(e1370); rbr-2(tm1231)* H3K4me3/  *daf-2(e1370); rbr-2(tm1231)* H3K4 | 0.395 | **0.282** |
| *daf-2(e1370); spr-5(by134)* H3K4me3/  *daf-2(e1370); spr-5(by134)* H3K4 | 0.189 | **0.064** |

**Table S4. Quantification of H3K4me2 compared to total H3K4 levels.**

| Strains | ratio replicate 1  H3K4me2/H3K4 | ratio replicate (in paper)  H3K4me2/H3K4 |
| --- | --- | --- |
| | N2 H3K4me2/N2H3K4 | | --- | | 0.705 | **0.329** |
| *daf-2(e1370)* H3K4me2/  *daf-2(e1370)* H3K4 | 0.591 | **0.243** |
| *daf-2(e1370);rbr-2(ok2544)* H3K4me2/  *daf-2(e1370);rbr-2(ok2544)* H3K4 | 0.359 | **0.127** |
| *daf-2(e1370); rbr-2(tm1231)* H3K4me2/  *daf-2(e1370); rbr-2(tm1231)* H3K4 | 0.191 | **0.121** |
| *daf-2(e1370); spr-5(by134)* H3K4me2/  *daf-2(e1370); spr-5(by134)* H3K4 | 0.792 | **0.306** |

**Table S5. Dauer formation and L1 or L2 larval arrest of progeny from *daf-2(e1370),* H3K4 demethylase mutants that were shifted from 15°C to either 20°C or 25°C as L4 larvae**

| strain | Temp  °C | L1 ^a^ | L2 ^a^ | dauer ^a^ | adults ^a^ | Total progeny scored (trials) |
| --- | --- | --- | --- | --- | --- | --- |
| *daf-2(e1370)* | 20 | -- | -- | 48.1 +/- 5.0 | 51.9 +/- 5.0 | 818(7) |
| *daf-2(e1370); rbr-2(ok2544)* | 20 | -- | -- | 24.2 +/- 2.1 ^b^ | 75.9 +/- 2.1 | 1113(11) |
| *daf-2(e1370); rbr-2(tm1231)* | 20 | -- | -- | 57.4 +/- 16.0 ^c^ | 42.6 +/- 16.0 | 114(4) |
| *daf-2(e1370); spr-5(by134)* | 20 | -- | -- | 4.0 +/-1.7 ^b^ | 96.0 +/- 1.7 | 961(7) |
| *daf-2(e1370)* | 25 | 4.4 +/- 0.8 | 1.0 +/- 1.0 | 94.7 +/- 0.9 | 0 | 234(6) |
| *daf-2(e1370); rbr-2(ok2544)* | 25 | 4.3 +/- 0.3 | 1.1 +/- 0.7 | 94.6 +/- 0.9 | 0 | 233(4) |
| *daf-2(e1370); rbr-2(tm1231)* | 25 | 15.9 +/- 2.3 | 5.4 +/- 2.1 | 78.7 +/- 3.6 | 0 | 268(10) |
| *daf-2(e1370); spr-5(by134)* | 25 | 5.4 +/- 2.8 | 2.6 +/- 1.3 | 92.0 +/- 3.3 | 0 | 248(7) |

^a^ mean percent +/- SEM; ^b^*P*<0.001 compared to *daf-2(e1370)*; ^c^ not significant compared to *daf-2(e1370)*

**Table S6. Dauer formation and L1 or L2 larval arrest of progeny from *daf-2(e1370),* H3K4 demethylase mutants that were shifted from 15°C to either 20°C or 25°C as embryos**

| strain | Temp  °C | L1 ^a^ | L2 ^a^ | dauer ^a^ | adults ^a^ | Total progeny scored(trials) |
| --- | --- | --- | --- | --- | --- | --- |
| *daf-2(e1370)* | 20 | -- | -- | 7.8 +/- 4.1 | 92.2 +/- 4.1 | 123(2) |
| *daf-2(e1370); rbr-2(ok2544)* | 20 | -- | -- | 9.6 +/- 1.9 ^b^ | 90.4 +/- 1.9 | 272(4) |
| *daf-2(e1370); rbr-2(tm1231)* | 20 | -- | -- | 72.4 +/- 7.6 ^c^ | 27.7 +/- 7.6 | 37(2) |
| *daf-2(e1370); spr-5(by134)* | 20 | -- | -- | 0 ^d^ | 100 | 79(2) |
| *daf-2(e1370)* | 25 | 0 | 0 | 100 | 0 | 113(2) |
| *daf-2(e1370); rbr-2(ok2544)* | 25 | ND | ND | ND | ND |  |
| *daf-2(e1370); rbr-2(tm1231)* | 25 | ND | ND | ND | ND |  |
| *daf-2(e1370); spr-5(by134)* | 25 | 1.8 +/- 1.8 | 2.1 +/- 2.1 | 96.1 +/- 2.3 ^d^ | 0 | 84(4) |

^a^ mean percent +/- SEM; ^b^ not significant compared to *daf-2(e1370)*; ^c^ *P*<0.05 compared to *daf-2(e1370)*; ^d^ *P*<0.5 compared to *daf-2(e1370)*
